# Congenital orbital teratoma presenting as microphthalmos with cyst

**DOI:** 10.4103/0301-4738.57152

**Published:** 2009

**Authors:** Usha Singh, Aparna Subramanian, Amanjeet Bal

**Affiliations:** Department of Ophthalmology

**Keywords:** Microphthalmos with cyst, orbital teratoma

## Abstract

We report a rare case of non-communicating large orbital cyst with microphthalmos which was surgically separated from the globe and excised. Histopathology reported it to be a teratoma. Congenital cystic teratoma should be a part of the differential diagnosis in an infant presenting with a clinical picture of microphthalmos with orbital cyst, in view of the different management required.

A congenital orbital teratoma is an extremely rare benign tumor[1,2] which contains a wide diversity of tissues derived from two or three germ cell layers that are foreign to the orbit.[3] The clinical presentation of primary orbital teratoma is that of an extreme unilateral proptosis with forward displacement of a developmentally intact globe by a large orbital tumor of variegated consistency in a newborn.[4] We report an unusual case of congenital orbital cystic teratoma that presented similar to a microphthalmos with cyst, not reported earlier.

## Case Report

**Figure 1:**
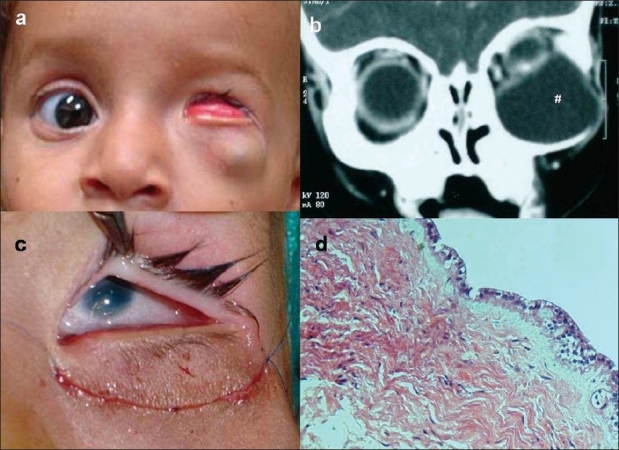
Cystic teratoma presenting with microphthalmos, (a) Clinical photograph, (b) Computed tomography showing a large cystic lesion occupying the inferior orbit and displacing the globe superiorly, (c) Intraoperative picture showing microphthalmos, (d) Cyst wall lined by cuboidal to columnar lining epithelium

A seven-month-old female child presented with history of mass lesion in the left lower lid since birth which was progressively increasing in size. Examination revealed a swelling in the lateral two-third of left lower lid, bluish in color and measuring 3 × 3 cm. The swelling was soft, fluctuant, with ill-defined borders and the surface had engorged blood vessels. It was nontender and did not increase in size on crying. The lower tarsal conjunctiva was everted and mechanical ectropion was visible [Fig. 1a]. The right eye was normal. Systemic examination revealed a ventricular septal defect and patent ductus arteriosus. Computed tomography (CT) scan revealed an enlarged orbit with no bone erosion [Fig. 1b]. The cystic lesion occupied most of the orbit displacing the microphthalmic globe and extraocular muscles superiorly. Neuroimaging excluded any communication with the globe or central nervous system. Anterior inferior orbitotomy with total cyst excision was done. the superior part of the cyst wall was found merged with the inferotemporal aspect of the sclera. Using gentle blunt and sharp dissection under the microscope, the cyst could be separated from the globe without breaching its integrity. The eye ball was found to be microphthalmic [Fig. 1c]. Corneal diameter measured 5 × 6 mm.

**Figure 2:**
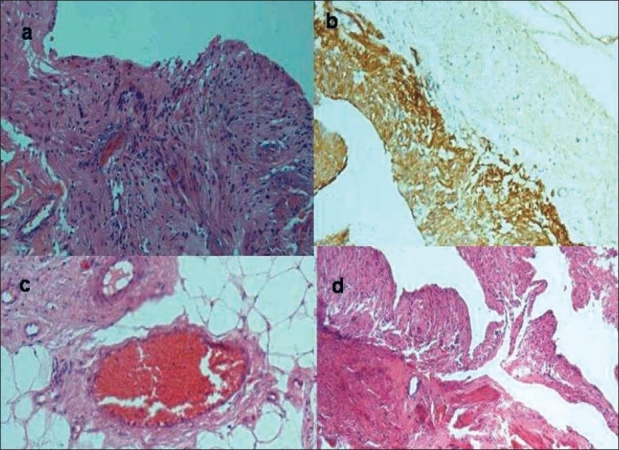
Histopathology of the cystic teratoma, (a) Neuroglial tissue, (b) GFAP highlighting neuroglial tissue, (c) Adipose tissue with blood vessels, (d) Bundles of smooth muscle in cyst wall

The specimen consisted of a flattened cyst like structure measuring 2.5 cm in diameter. It had congested outer surface and smooth inner surface. There were large areas containing multiple cysts. Microscopic examination revealed tissue derived from all three germ layers. The wall of the cyst was lined focally by tall columnar epithelium [Fig. 1 d]. There was neuroglial tissue which was confirmed by the glial fibrillary acidic protein (GFAP) immunostain. Surrounding areas showed presence of fat, mature smooth muscle and nerve bundles. The tissues were mature, without mitotic activity [Fig. 2a–d].

Postoperative evaluation revealed a microphthalmic eye with no light fixation. The ocular movements were present with some limitation in abduction only. Microcornea was opaque with no view of anterior segment details. Patient's parents were explained about the poor visual prognosis and the need for ocular prosthesis. One year postoperatively there was no recurrence.

## Discussion

The clinical presentation of primary congenital orbital teratoma is spectacular. Extreme proptosis at birth, coupled with a developmentally intact globe and an orbital mass having a variegated appearance and consistency, is essentially diagnostic of primary orbital teratoma.[5] Clinically it is difficult to distinguish other benign and malignant neoplasms from teratoma. The differential diagnoses for cystic anomalies include microphthalmia with cyst, congenital cystic eye, microphthalmia with cystic teratoma, ectopic brain tissue and meningoencephalocele. Microphthalmos with cyst is distinguished on the basis of a small globe in conjunction with a communicating channel between it and an attached cyst.[6] Rarely, the congenital colobomatous microphthalmos with orbital cyst may not communicate with the eye and or the central nervous system. Our patient presented with an orbital cyst and clinically nonvisible microphthalmic eye simulating the clinical presentation of a microphthalmos with cyst. Given the above clinical situation, diagnosis of teratoma was least considered. Intraoperative examination revealed microphthalmic eye along with an orbital cyst.

Histopathologically, teratomas are benign growths of mature and immature tissues characterized by aggregations of well-differentiated tissue of diverse germ cell origin.[7] The predominant germ cell type observed in orbital teratomas is surface ectoderm, producing squamous epithelial-lined cysts filled with keratin, adnexal structures such as hair follicles, and sweat glands. Neuroectodermal structures include grey and white matter of the brain, primitive neural tubes, ocular primorida, choroid plexus, and ganglia. Mesoderm is the next most common germ cell layer, represented by such tissues as muscle, bone cartilage, and fat. Endoderm is the least common and usually manifests as gastrointestinal tissue or as cysts lined by respiratory type pseudo-stratified columnar epithelium. Whereas the microphthalmos with cyst is a two-layered structure. The inner layer is composed of a gliotic neuroretinal tissue that may show retinal architecture, photoreceptor differentiation, or rosette formation.[8] The outer layer consists of vascularized connective tissue and may occasionally contain cartilage. In our case the cystic lesion had derivatives from all three germ cell layers and as per Duke-Elder's,[9] one of the five-point classification for orbital teratoma [Table 1], a tumor consisting of all three germinal layers would be a teratoma.

**Table 1:** Duke-Elder's classification of teratomata in the orbit[9]

A complete fetus implanted in the orbit A portion of a second fetus in the orbit A tumor consisting of all three germinal layers Tumors containing representatives of two germinal layers only Tumors containing representatives of one layer only

In conclusion, though congenital orbital teratoma is characteristically associated with a developmentally normal globe, rarely the globe could be microphthalmic. Orbital teratoma must be considered in the differential diagnosis of a child presenting with an orbital mass and a small or unrecognizable eye. In such cases prompt surgical excision is the treatment of choice.
